# Impact of temporal resolution on cardiac phase-resolved oxygen-sensitive myocardial steady-state free precession imaging

**DOI:** 10.1186/1532-429X-11-S1-P178

**Published:** 2009-01-28

**Authors:** Xiangzhi Zhou, Richard Tang, Rachel Klein, Sotirios A Tsaftaris, Debiao Li, Rohan Dharmakumar

**Affiliations:** 1 Department of Radiology, Northwestern University, Chicago, IL USA; 2 Department of Electrical Engineering and Computer Science, Northwestern University, Evanston, IL USA

**Keywords:** Image Quality, Regional Signal Difference, Late Diastole, Myocardial Signal, Late Systole

## Introduction

Cardiac phase-resolved imaging studies that are used in the assessment of cardiac function are performed with a temporal resolution (T_RES_) of approximately 50 ms to mitigate the effects from cardiac motion and flow. To date, there has been minimal interest on the characterization of myocardial signal intensities from cine images. Steady-state free precession based cardiac phase-resolved blood-oxygen-level-dependent (CP-SSFP BOLD) imaging is a relatively new method for identifying myocardial oxygen abnormalities on the basis of regional signal differences. For reliable assessment of oxygenation changes, it is imperative to ensure that acquisitions enable robust image quality. We hypothesize that T_RES_ plays a significant role on CP-SSFP image quality and that, in particular, myocardial signal characteristics disintegrate with elevations in T_RES_.

## Methods

Dogs were used to test the hypothesis under controlled conditions. Animals (n = 4) were anesthetized and their heart rate was monitored with ECG (R-R interval = 710 ms–780 ms). Multiple breath-held acquisitions (20–40 s) were performed in each animal, interrupted by 2–3 minute rest, ensuring that the heart rate remained relatively constant between acquisitions. 2D balanced-SSFP imaging was prescribed in the cine mode to study the effects of T_RES_ on short-axis mid-left-ventricular images of the myocardium in a clinical 1.5 T scanner. The scan parameters were: in-plane resolution = 1.2 × 1.2 mm^2^, TR/TE = 3.5 ms/1.75 ms (conventional cine SSFP) and 6.0 ms/3.0 ms (CP-SSFP BOLD), flip angle = 70°, NEX = 1, segments/cardiac phase were changed to obtain different T_RES_ (10 ms to 200 ms). This study was repeated 2–3 times with a two-day interval for each animal. In total 10 studies were performed. Two indices were used to quantify the myocardial signal characteristics obtained with cine SSFP images at different T_RES_: (1) *Myocardial Signal Inhomogeneity Index* (MSI), defined as the standard deviation of the LV myocardial signal intensity; and (2): *Transmural Heterogeneity Index* (THI), defined as the minimum pixel intensity difference between pixels along a line perpendicular to the blood-muscle interface in the LV chamber. The global THI was calculated by sweeping the line along the interface for 360° and averaging the THI for each 1° increment. Results were averaged across all studies for late systole(LS) and late diastole(LD). Note that MSI measures the signal variation throughout the myocardium and THI measures the image quality permitting reliable delineation of the endocardial border from blood.

## Results

**Figure 1:**
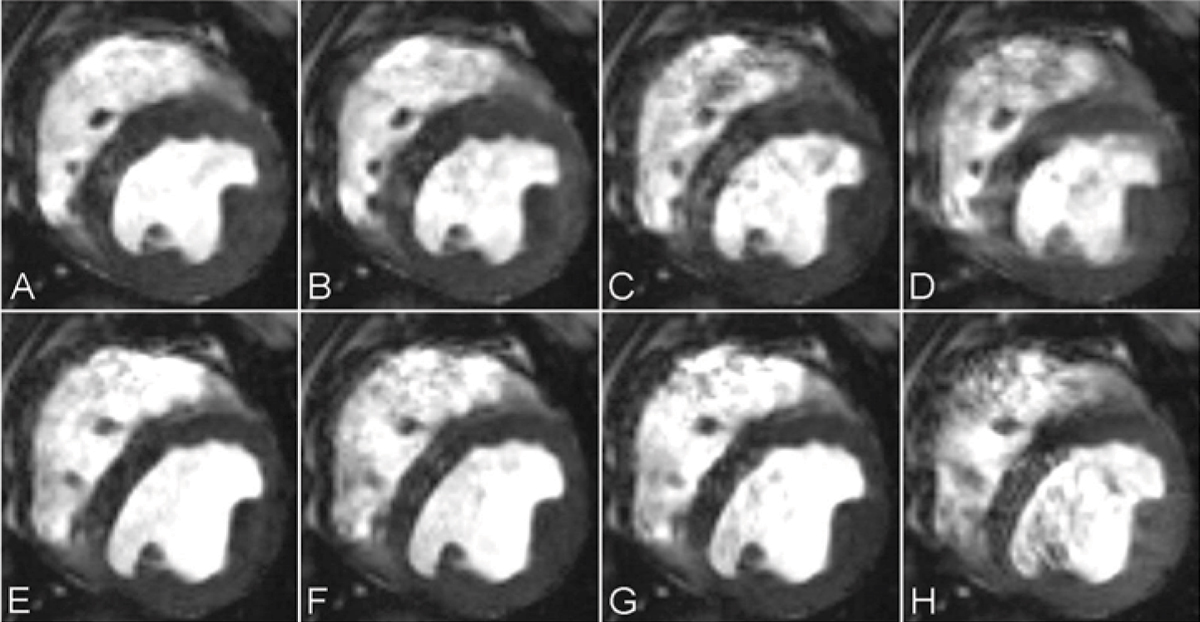
**Short axis SSFP images (TR = 6.0 ms) of med ventricle with different T**_**RES**_ **A,E:18 ms; B,F: 42 ms; C,G: 84 ms; and D,H: 204 ms (upper row from LS and lower row from LD)**. Note that as T_RES_ increases the signal inhomogeneity increases and endocardial delineation becomes more difficult as signal from the blood pool blends in with the myocardial signal, likely due to motion.

**Figure 2:**
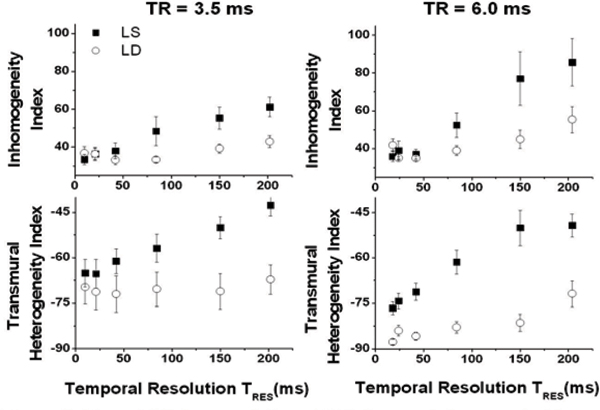
**Mean MSI (upper plot) and THI (lower plot) computed from LS (squares) and LD (circles) images as a function of T**_**RES**_ **for TR = 3.5 ms (left) and 6.0 ms (right)**. Note that both mean MSI and THI increase with increasing T_RES_.

Figure 1 shows short-axis SSFP images with different T_RES_ (10 ms, 42 ms, 80 ms and 202 ms from left to right obtained at TR = 6.0 ms). The upper row images are from LS and the lower row images are from LD. Numerical results from MSI (upper plot) and THI (lower plot) computations from the LS and LD images as a function of T_RES_ are shown in Figure 2. On average, MSI and THI are directly related to T_RES_ at LS and LD (for TR = 3.5 and 6.0 ms), albeit the rate of change of the indices at LS is much greater than at LD. In particular, results showed that, at TR = 6.0 ms, for T_RES_>42 ms, MSI and THI are significantly greater than with T_RES_ = 18 ms (t-test, p < 0.01).

## Conclusion

Reliable image quality is critical for accurate detection of changes in myocardial oxygenation. This study investigated the impact of T_RES_ on two important features of CP-SSFP BOLD images: (1) myocardial signal variations, (2) endocardial blur. Findings show that MSI and THI are strongly influenced by T_RES_. In particular, with both conventional cine SSFP (TR = 3.5 ms) and CP-BOLD SSFP (TR = 6 ms) imaging, the image quality diminishes with increasing T_RES_. Also, for any given T_RES_, the reduction in image quality is significantly greater at systole than at diastole. We conclude that for reliable detection of myocardial oxygenation on the basis of CP-SSFP BOLD imaging, it is necessary to keep T_RES_ as short as possible. These findings remain to be validated in humans.

